# The Small Medical Colleges

**Published:** 1908-01

**Authors:** 


### SELECTIONS AND ABSTRACTS.

### THE SMALL MEDICAL COLLEGES.

A notable introductory address, entitled "Educational Democracy," was delivered at the opening of the seventy-seventh session of the Albany Medical College by Dr. Willis G. Tucker, professor of chemistry and toxicology in the college. It is published in the November number of the *Albany Medical Annals*. "Our smaller colleges," says Professor Tucker, "deserve and should receive fuller recognition and better support." We agree entirely with the speaker. We have never been able to discover that any physician was at all more excellent by reason of his having been graduated by a great university, and we are heartily tired of having it dinned into our ears that such and such a man must be superior on account of the greater number of years spent by him in undergraduate study. We always feel like retorting that it takes longer to gild a dolt than to educate a bright man.

We have heard too much of late years about the necessity of lengthening the medical course and raising the preliminary requirements. As regards the preliminaries, there are, says Professor Tucker, those who would "reduce everything to a strict numerical expression. They pursue their investigations with a foot rule and hourglass." They measure the floor spaces of the recitation rooms and laboratories in which the candidate has been instructed, and appraise the cash value of the apparatus employed. As well estimate a man by his vocabulary, as the faddists generally do! Our forefathers had no enforced preliminaries, yet there was far less illiteracy in the medical profession fifty years ago than there is to-day. In spite of the present requirements, Professor Tucker says: "Men who spell wretchedly and make bad work with simple arithmetical calculations still enter, armed with State credentials, and last year in this school more first year men were conditioned than in any previous year within my recollection." He adds that he is not inclined to believe that the average reputable American physician to-day is in any recognizable degree superior to his predecessor of a quarter of a century ago *as a result* of State supervision.

Where were our great men of the past in medicine graduated? Who cares to know what the institutions are or were (for some of them are defunct) upon which credit has been shed by such men as Rush, Bard, the Warrens, the Nathan Smiths, Bartlett, Jackson, Drake, McDowell, Hodgen, Pancoast, Gross, Sims, Eve, March, Bontecou, Mott, Parker, Clark, Bigelow, Hodge, Meigs, Thomas, Van Buren, Flint and Barker? We have had enough of snobbery hinging on the source of the degree or on the State requirements for the license. Let us encourage the less pretentious of our schools.—Editorial, New York Medical Journal, Nov. 16th. 1907.

